# Circular RNAs: novel noncoding players in male infertility

**DOI:** 10.1186/s41065-024-00346-8

**Published:** 2024-11-18

**Authors:** Emad Babakhanzadeh, Fakhr-Alsadat Hoseininasab, Ali Khodadadian, Majid Nazari, Reza Hajati, Soudeh Ghafouri-Fard

**Affiliations:** 1 Department of Medical Genetics, Shahid Beheshti University of Medical Sciences, Tehran, Iran; 2 Department of Medical Genetics, Shahid Sadoughi University of Medical Sciences, Yazd, Iran; 3 Department of Medical Immunology, Shahid Sadoughi University of Medical Sciences, Yazd, Iran

**Keywords:** Non-coding RNAs, circRNA, Male infertility, Spermatogenesis

## Abstract

Infertility is a global problem being associated with emotional and financial burden. Recent studies have shown contribution of a group of non-coding RNAs, namely circular RNAs (circRNAs) to the etiology of some infertility conditions. CircRNA are transcribed from exons and form a circular RNA molecule, being abundant in eukaryotes. Traditionally classified as non-coding RNA, these transcripts are endogenously produced through either non-canonical back-splicing or linear splicing, typically produced from precursor messenger ribonucleic acid (pre-mRNA). While during the canonical splicing process the 3’ end of the exon is joined to the 5’ end of the succeeding exon to form linear mRNA, during backsplicing, the 3’ end to the 5’ end of the same exon is joined to make a circular molecule. circRNAs are involved in the regulation of several aspects of spermatogenesis. They appear to influence how stem germ cells grow and divide during the sperm production process. Malfunctions in circRNA activity could contribute to male infertility issues stemming from abnormalities in spermatogenesis. In the current review, we highlight the exciting potential of circRNAs as key players in the male fertility.

## Introduction

Infertility is the inability of a couple to conceive after an entire year of consistent attempts [1]. Around 10–20% of couples worldwide are affected by this problem [2, 3]. Although either partner can cause infertility, male factors account for 30–50% of cases [4, 5]. Despite this, up to 25% of couples are never tested, highlighting the need for a more comprehensive testing [6–8]. Since a significant portion (up to 10%) of the male genome is dedicated to reproductive functions, it is understandable that genetic factors play a major role in male infertility [9, 10]. Research shows that the likelihood of genetic abnormalities increases with a low sperm count [11–13].

Recent improvements in high-throughput sequencing techniques (such as whole-exome and whole-genome sequencing, RNA sequencing) have provided new insights into male infertility [14, 15], having uncovered the involvement of non-coding RNAs (ncRNAs) as crucial regulators of spermatogenesis.

The cell needs proteins to properly execute its functions. Proteins are synthetized in cytoplasmic ribosomes, which read the sequence of a molecule named messenger ribonucleic acid (mRNA). The genetic information is contained in the DNA (deoxyribonucleic acid) sequence, which is a double stranded molecule (in the form of a helix). Certain DNA sequences, named genes, correspond to proteins. Through a step named transcription, the DNA sequence of a gene is copied into a single stranded (linear) molecule named messenger RNA (mRNA). The copied DNA gene contains sequences named introns (non-coding sequences) and exons (coding sequences), and the transcript is named heterogeneous mRNA (or pre-mRNA). During the process of transcription, small nuclear RNAs (snRNA) act as enzymes and remove introns and fuse exons (splicing of the mRNA), whereas other snRNA add a protective cap at the 5´end and a protective poly-A tail (repetitive regions of adenines) at the 3´end of the mRNA. During the process of intron excision (splicing), the intronic sequence of each intron forms a lace-like RNA fragment named lariat. Usually these fragments are degraded in the nucleus. The mRNA molecule then passes through nuclear pores and enters the cytoplasm. There, the mRNA is translated (step of translation) into proteins with the aid of ribosomes. Splicing can also occur at different intro-exon margins, giving origin to mRNAs with a different number of exons. In these cases, a same DNA gene sequence can give origin to different proteins, a process named alternative splicing.

Non-coding RNAs are a heterogeneous group of small nuclear RNAs (snRNAs) that, by definition, are not translated into proteins in the cytoplasm. However, ncRNAs act as crucial regulators of several biological processes within numerous cell types and tissues, and their dysregulation is involved in diseases [16–18].

In particular, three major classes of ncRNAs have emerged as important players: long non-coding RNAs (lncRNAs), circular RNAs (circRNAs) and microRNAs (miRNAs) [19–26]. Interestingly, research suggests that many of these ncRNAs are not only involved in the development of male infertility [27], but may also be biomarkers [28]. CircRNAs are single-stranded covalently closed RNA molecules that have attracted attention due to their diverse roles in gene regulation [29]. They are formed by a non-canonical splicing process, known as back-splicing, in which the 5’ and 3’ ends of a linear mRNA are joined to form a closed loop [30, 31].

CircRNAs have diverse functions in cells, including acting as miRNA sponges (inhibition of the miRNA action) to modulate miRNA activity, regulating protein binding and gene transcription, and possibly having a coding function [32, 33]. They are more stable than linear RNAs since they lack the 3’ poly(A) tails and 5’ end caps, making them more resistant to degradation [34]. Their expression and function in male infertility is also being elucidated [35]. However, further research is needed to clarify the mechanisms underlying the regulation of circRNAs in male fertility and to explore their potential as diagnostic biomarker or therapeutic target [36].

## Biogenesis, diversity and functional roles of circRNAs

**Fig. 1:**
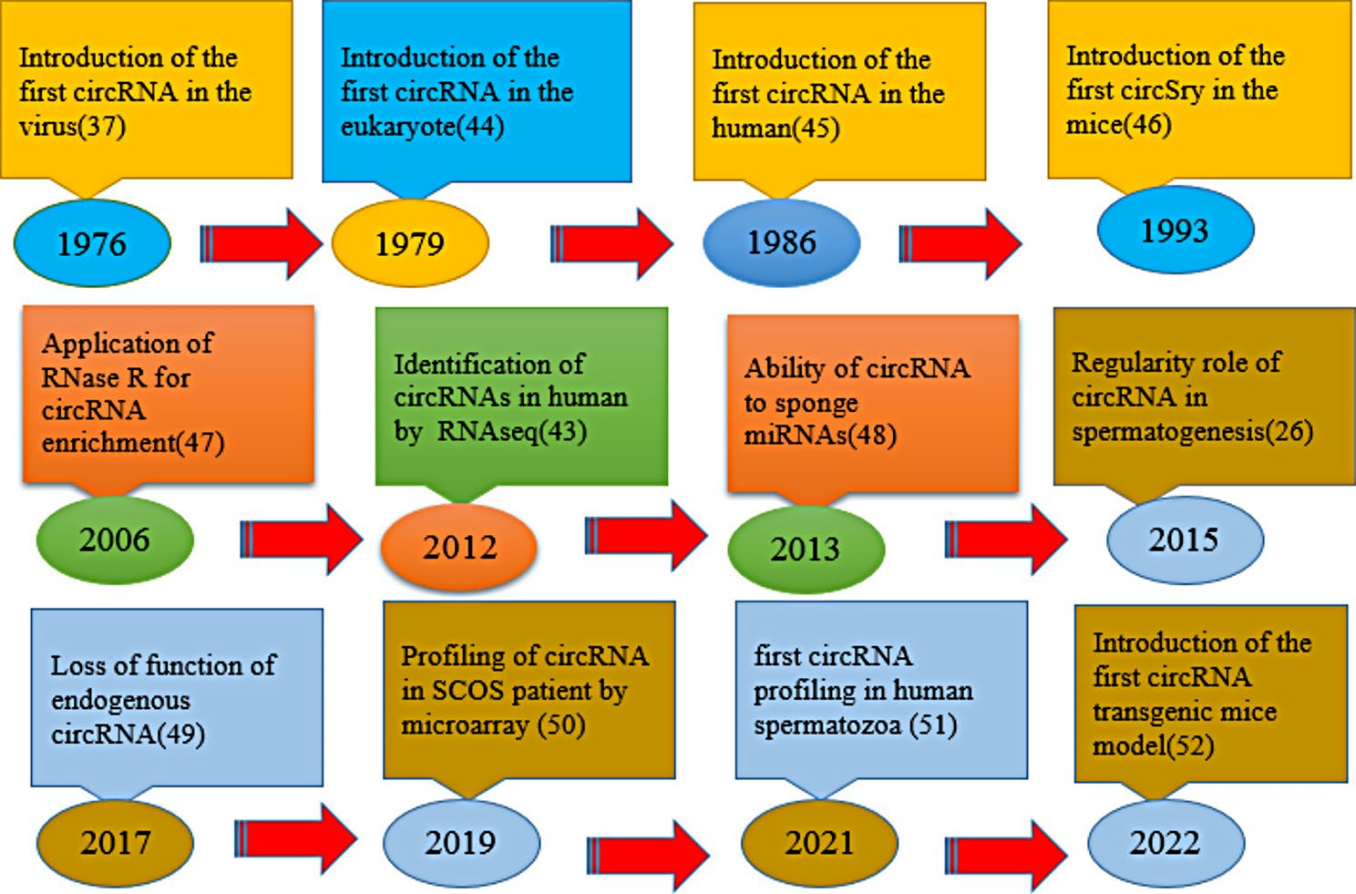
Timeline of progress in circRNA; from discovery to role in male fertility

While circRNAs were first discovered in hepatitis delta virus (HDV) in 1976 [37], their abundance and function remained largely unexplored due to their limited detection and association with splicing defects. A turning point came in 2013 with the publication of Hansen et al. [38] which reignited scientific interest in circRNAs (Fig. 1). Subsequent research revealed a vast landscape of thousands of circRNAs in plants [39] and humans [40] whose functions go far beyond mere splicing artifacts [41]. In particular, circRNAs in plants were shown to act as negative regulators of their parental genes [32], highlighting their potential for controlling gene expression [42]. Today, RNA sequencing data indicate the impressive presence of nearly 100,000 circRNAs expressed in humans, pointing to their widespread and crucial role in biological processes beyond those originally envisioned [43].

CircRNAs are a recently discovered class of non-coding RNAs characterized by their covalently closed, circular structure. In contrast to their linear counterparts, circRNAs lack the 5’ caps and 3’ poly(A) tails, which gives them increased stability against exonucleolytic degradation (degradation of RNA molecules con be performed inside the sequence (endonucleases) or at the ends of the molecule (exonucleases) [44]. This unique property positions circRNAs as potential regulators of gene expression (the expression of genes into proteins by means of transcription into RNA molecules, can be activated or inhibited, which is named gene regulation) with effects on various biological processes and disease states. Several mechanisms contribute to the circRNA biogenesis. These mechanisms include: Exon skipping (during splicing of pre-mRNA, downstream exons bypass upstream exons and assemble into a circular RNA with skipped exons); direct back-splicing (this process involves the direct ligation of non-contiguous exons or introns, resulting in a circular RNA molecule); and debranching of intronic lariats (rearrangement and debranching of the lariat intermediate formed during splicing can generate circRNAs with conserved introns). The mechanism used in each case can influence the functional properties and regulatory potential of the resulting circRNAs [45–50].

**Fig. 2:**
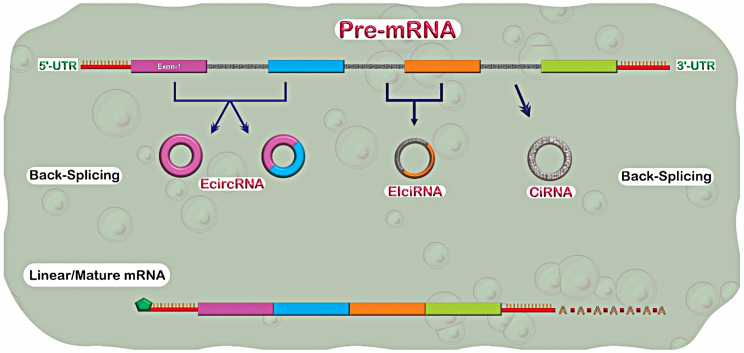
Different subtypes of circRNAs

CircRNAs have different characteristics and perform different biological functions [51, 52]. There are different subtypes classified according to their composition, including exonic circRNAs (ecRNAs), which contain only exons, exonintronic circRNAs (EIciRNAs) with exons and introns, and intronic circRNAs (ciRNAs) (Fig. 2) [53]. The functional roles of circRNAs include (a) miRNA sponges: circRNAs can competitively bind miRNAs, preventing their interaction with target mRNAs and thereby regulating gene expression (translation inhibition) [54]; (b) protein scaffolding: some circRNAs act as platforms for protein binding, facilitating the assembly of protein complexes and modulating protein function [55]; (c) transcriptional regulation: circRNAs can interact with RNA polymerase II (enzyme that transcribes DNA to mRNA) or other transcriptional regulators to influence gene transcription; (d) alternative splicing: circRNAs can modulate alternative splicing patterns by competing with pre-mRNA for splicing factors [33, 56, 57].

### Spermatogenesis process

Spermatogenesis is a complex, highly regulated process, which occurs in the germinal epithelium of the seminiferous tubules (ST) in testicles that produces haploid germ cells (spermatozoa) [58]. The ST epithelium contains the somatic supportive and nutrient Sertoli cells (SC) and germ cells. The epithelium is covered by a basal lamina that contains fibromyoid cells (peritubular cells). In the interstitium (loose connective tissue) resides nerves, lymphatics, blood vessels and Leydig cells. Attached to the basal membrane reside stem germ cells (spermatogonia A-dark). Through mitosis, these originate A-pale spermatogonia and then B-spermatogonia. After that stage, germ cells enter meiosis (spermatocytes), where chromosomes replicate (primary spermatocyte Leptotene stage), condense and form pairs between homologous (primary spermatocyte zygotene stage). At the primary spermatocyte pachytene stage occur homologous recombination (crossing-over), with exchange of chromosome regions between homologous chromosomes (except with the sexual chromosomes), which have essential role in accurate chromosome segregation and establishment of new combinations of parental alleles and thus secure population diversity. Thereafter, the cell enters the first meiosis (separation of chromosomes, each with two replicated chromatids), with formation of secondary spermatocytes. These enter the second meiosis (separation of chromatids) with formation of round (haploid) spermatids (add Ref and scheme). Sertoli cells are attached to the basal lamina and present basal membrane receptors for follicle-stimulating hormone (FSH) and testosterone. Binding of the hormones activates gene expression. It is SC that release estrogen (contains aromatase that converts testosterone), androgen-binding protein (acts as a reservoir and transport molecule for testosterone), inhibins (suppresses FSH release), activins (activate FSH release), antiapoptotic agents, stem cell factor (stimulates spermatogonia differentiation), transcription factors, transferrin, ceruloplasmin, and nutrients to germ cells, in order to spermatogenesis proceed. The SC are also responsible for the establishment of an immunoprotective blood-testis-barrier and phagocytosis of apoptotic germ cells [59].

The human ejaculate contains a mixture of spermatozoa with different characteristics such as shape, size and motility [60]. After being formed, testicular spermatozoa with the accompanying ST fluid are expelled from the ST into the rete-testis by means of ST wall contractions. In the rete-testis, the continuous ST fluid movement, push spermatozoa through efferent tubules until they reach the head (caput) of the epididymis. Here, the ST fluid is reabsorbed and spermatozoa concentrate. In the middle region (corpus) of the epididymis, exosomes released from the epithelial cells fuse with the spermatozoa cytoplasmic membrane, changing its protein, phospholipid and cholesterol composition. Additionally, the sperm chromatin attains its final condensation phase through establishment of bisulfite bonds between protamines (sperm maturation) [61]. Spermatozoa are stored in the last epididymis region (cauda). Testicular and epididymal spermatozoa are immotile. However, if aspirated and in contact with air, the loss of CO2 pressure enables an increase in cytoplasmic pH and activates motility [62]. Without maturation (epididymis transit) and capacitation (uterine cavity transit), spermatozoa are not able to fertilize the oocyte, unless directly microinjected into the oocyte cytoplasm [63]. At ejaculation, the peristaltic contractions of the vas deferens expel spermatozoa towards the exterior [64]. The ejaculate contains about 10% of spermatozoa and accompanying fluid, 60% of seminal vesicle secretions and 30% of prostate and bulbourethral gland secretions, which compose the seminal fluid [65]. The seminal fluid (or seminal plasma) contains numerous protective ions and proteins, which has been used for discovering several markers of spermatozoa quality (sperm number, morphology, motility and DNA integrity), including snRNAs [66].

High-quality spermatozoa (referred to here as “A sperm”) exhibit features that are critical for fertilization: normal morphology, efficient motility and maximal DNA integrity (maximal DNA compaction and minimal DNA fragmentation). Conversely, low quality sperm (“B-sperm”) are characterized by high sperm DNA fragmentation, abnormal sperm concentration, morphology and/or motility [67]. In infertility treatments, techniques such as assisted reproductive technology (ART) techniques aim to select A sperm to improve fertilization success.

Spermiogenesis is the final stage of spermatogenesis, in which a round haploid spermatid differentiated into testicular spermatozoa [68–70]. This complex process involves several important steps, namely, acrosomal vesicle and flagellum formation, and nuclear elongation. Excedentary cytoplasm is shed and reabsorbed by Sertoli cells. The acrosomal vesicle is formed from vesicles originated from the Golgi (or dictyosome). They fuse into a single large vesicle that appose to the future upper nuclear pole and begins to spread along the nuclear envelope until covers the upper 2/3 of the nucleus. This acrosomal vesicle cap is a modified lysosome that contains inactivated hydrolytic enzymes. When the sperm surface membrane receptor binds to oocyte zona pellucida glycoproteins, the acrosomal vesicle suffers exocytosis (acrosomal reaction); the acrosomal enzymes are then activated, and used to perforate the zona pellucida, enabling sperm entry into the perivitelline space where a sperm membrane receptor binds to an oocyte membrane receptor, declanching gamete membrane fusion and total sperm incorporation into the oocyte (the sperm membrane becomes part of the oocyte membrane). The sperm flagellum begins to be synthesized in the cytoplasm but later at the round spermatid stage begins to be exteriorized. The distal centriole that originates the axoneme will be later transformed into a centriole-like appendix. The axoneme becomes surrounded by fibers, which, at the initial part of the flagellum, the midpiece, are surrounded by an ubiquitinated helix of around 70 mitochondria. The round nucleus, with the aid of microtubules rows (manchette), is progressively elongated. During this time, nuclear histones are replaced by protamines, which can much more compact the sperm chromatin [71]. The proximal centriole remains attached to the base of the sperm nucleus. When inside the oocyte cytoplasm, both sperm centrioles originate the zygote aster, as the oocyte chromosome spindle is devoid of centrioles (lost during oogenesis) [72–76].

In the spermiation stage, SC actively release testicular spermatozoa into the ST lumen [77]. In humans, the entire process of sperm production (spermatogenesis) takes around 64 days [78]. New waves of sperm development can begin even before the previous cycle is completed. Groups of developing germ cells at different stages are organized in different zones within the ST epithelium. Spermatogenesis in humans is divided into six premeiotic stages (I-VI) and six stages of spermatid (post-meiotic) development, which take place in a non-uniform pattern within the ST. Each new generation of germ cells (originating from spermatogonia stem cells) is linked to the development of the previous generation, thus ensuring a continuous production process [77, 79–84].

### CircRNAs and spermatogenesis

CircRNAs are becoming increasingly important in the context of sperm quality; they have been found in the seminal fluid and testes of mammals and in human sperm in both healthy and diseased humans. The circRNA in sperm is a unique “sperm RNA code” that can be used to assess sperm quality and thus influence embryonic development [85–91].

To investigate the possible role of circRNAs in spermatozoa quality, researchers compared the expression patterns of circRNAs in two groups of human spermatozoa: high-quality (A SPZ) and low-quality (B SPZ). Using a circRNA microarray technique, authors identified 148 circRNAs with significantly different expression levels between the two sperm quality, with 91 upregulated and 57 downregulated genes in the low-quality sperm compared to the high-quality sperm group [92]. In 2023, a study looked at the role of circRNAs in goat reproduction. Their microarray result showed the importance of circRNA_07172, circRNA_04859, circRNA_07832, circRNA_00032 and circRNA_07510 in the development of testis and spermatogenesis [93].

Bahlibi et al. deciphered the intricate function of circRNAs in the testes of Holstein bulls and revealed their potential role as regulators of spermatogenesis. In their study, 3,032 differentially expressed circRNAs (DEcircRNAs) were identified, together with 683 miRNAs and 14,081 mRNAs. This vast network of ncRNAs appeared to play a crucial role in controlling key steps of spermatogenesis, including cycle checkpoints, chromosome and cytoplasmic segregation, sperm tail formation and ultimately sperm motility [94]. In another experiment, 2326 DEcircRNAs were found to be involved in the regulation of spermatogenesis and cilia motility [95]. These results provide exciting clues to the intricate regulatory network orchestrated by circRNAs in testis development. Understanding how circRNAs are associated with specific processes such as spermatogenesis and sperm motility could pave the way for new strategies to improve male fertility and reproductive health.

The cannabinoid receptor CB1 plays a crucial role in spermatogenesis and sperm maturation in the epididymis. Researchers investigated the link between endocannabinoids, molecules naturally produced by the body and known to interact with CB1, and the circRNA found in sperm. In this study, a unique pathway independent of the competing endogenous RNA (ceRNA) network was identified: circLIMA1 interacted with a protein called and enters the sperm nucleus, where it regulates another protein, gelsolin (actin-binding protein), which affects actin organization. QKI is an RNA binding protein that is involved in the regulation of pre-mRNA splicing, transfer of mRNAs from the nucleus, protein translation, and mRNA stability. It is also involved in myelinization and oligodendrocytes differentiation. In mice lacking CB1, circLIMA1 levels in sperm are elevated, leading to inefficient degradation of actin in the nucleus. This suggests that endocannabinoids contribute to proper sperm shape and cellular maturation through their influence on circRNA cargo [96]. Since proper actin organization is critical for the fusion of the spermatozoon and oocyte nuclei during fertilization (at fertilization, the sperm nuclear chromatin decondenses and originates the male pronucleus; the activated oocyte enters the second meiosis and the female chromosomes become encircled by a nuclear envelope, thus originating the female pronucleus; the two sperm centrioles transform into active asters, and the microtubules irradiating by them polymerize until touching the female pronucleus nuclear envelope; at that time, aster microtubules begin depolimerization, moving the pronuclei to the center of the zygote (zygote, is the oocyte with two pronuclei and two polar bodies), becoming juxtaposed; at the site of juxtaposition, the nuclear envelopes of the pronuclei originate short expansions that intermingle; lamins are then phosphorylated, and the nuclear envelopes break down into small fragments; the chromosomes intermingle (homologue juxtaposition), and the first mitosis succeeds, originating an embryo with two blastomeres) [97, 98], circLIMA1 may also play a key role in regulating this process and ensuring successful embryonic development.

Cannabinoid receptors are present throughout the body, and are part of the endocannabinoid system [99]. Cannabinoid receptors are transmembrane proteins, whose cytoplasmic portion is coupled to the G-protein [100]. The G-protein is a guanine nucleotide-binding protein that acts as molecular switch inside cells, being thus are implicated in conveying signals from a range of stimuli outside a cell after binding a ligand to a receptor [101]. The endocannabinoid receptor type 1 (CB1) is a type of cannabinoid receptor found in the brain. It is activated by endocannabinoid neurotransmitters, such as anandamide, serotonin and melatonin, and by the psychoactive compound “THC”, found in the flowers of the cannabis plant [102]. CB1 receptors are G-coupled proteins (GPCRs) that, when activated, initiate an intracellular signaling cascade that affects neurotransmission [103]. They are encoded by the ***CNR1*** gene and their activation is mediated primarily by ∆9-tetrahydrocannabinol (THC), the main psychoactive compound found in Cannabis, as well as by endocannabinoids, which are cannabinoids naturally produced by the body, such as anandamide and 2-arachidnoylglycerol (2-AG) [104, 105]. CB1 is encoded by the *CNR1* gene, which is expressed both in the peripheral and central nervous systems [106]. It is activated by a range of endogenous cannabinoids and plant phytocannabinoids [107, 108]. The phytocannabinoid tetrahydrocannabivarin (THCV) is the antagonist of CB1 [109]. Inside the liver, induction of the CB1 receptor increases *de novo* lipogenesis [110].

Cannabinoid receptors have two subtypes, namely CB1 and CB2 [111]. Receptor CB1 is principally expressed in the central nervous system, but also in the lungs, liver and kidneys [112]. CB2 is principally expressed in the immune system, hematopoietic cells, and in the brain [113].

Cannabinoid receptors act through endocannabinoid-mediated depolarization-induced suppression of inhibition, a very common form of retrograde signaling. Through this process, depolarization of a particular neuron results in a decrease in GABA-mediated neurotransmission [114].

### The role of circRNAs in controlling function of mitochondria during spermatogenesis

Researchers discovered a newly identified protein, Rsrc1-161aa, which is encoded by a unique circular RNA molecule (circRsrc1) in mouse testes. In mice lacking circRsrc1, fertility was impaired due to reduced sperm count and motility, which was attributed to impaired energy production in their mitochondria. Experiments confirmed that circRsrc1 regulates mitochondrial function through its encoded protein. Further analysis revealed that Rsrc1-161aa interacts directly with another protein, C1qbp, and increases its ability to bind to mitochondrial mRNA (mtRNA). This interaction affects the assembly of mitochondrial ribosomes and the production of proteins that are critical for energy production (oxidative phosphorylation). In simpler terms, the study revealed a novel protein involved in sperm production and fertility, highlighting its role in regulating energy production in the mitochondria [115].

### CircRNAs linking male infertility and obesity

Obesity, a growing problem worldwide, is associated with male fertility problems [116]. However, the exact link between obesity and impaired sperm function remains unclear. In an experiment, Francesco et al. used mice fed a high-fat diet (HFD) as a model for obesity and oxidative stress. They investigated the effects of HFD on sperm morphology, motility and the presence of circRNAs in the sperm (sperm circRNAs). Key findings include: (i) HFD caused changes in sperm shape and motility, (ii) sperm from HFD mice exhibited altered circRNA profiles compared to healthy controls, (iii) some circRNAs were upregulated due to increased backsplicing activity within the sperm themselves, (iv) other circRNAs were downregulated due to inefficient production in the epididymis, a male reproductive organ, (v) two specific circRNAs, circADAM10 and circPCSK6, were identified as potentially involved in the response to oxidative stress. Their study suggests that obesity disrupts the machinery responsible for the production of circRNAs in both the sperm and the epididymis. This disruption could lead to changes in sperm function and contribute to fertility problems [117]. This study provides new insights into how obesity might affect male fertility by altering the circRNA load of sperm. Further research is needed to understand the specific functions of these circRNAs and their potential utility in the diagnosis or treatment of obesity-related male infertility.

### The role circRNAs in controlling heat shock proteins in the context of male infertility

Scientists have found that mice with impaired circBoule RNAs show reduced fertility and flies lacking these RNAs show impaired reproductive capacity under heat stress conditions. During spermatogenesis, circBoule RNAs in flies interact with heat shock proteins (HSPs), while their counterparts in mouse sperm bind to HSPA2. This interaction between circBoule RNAs and HSPs is also maintained in humans, where lower levels of circBoule RNAs circEx3-6 and circEx2-7 are associated with sperm motility problems. These results suggest that circBoule RNAs have conserved physiological functions in animals and may play a critical role in regulating male reproductive function under stress [118–120].

### CircRNAs and non-obstructive azoospermia

In the context of male infertility, obstructive azoospermia (OA) and non-obstructive azoospermia (NOA) are two different categories of azoospermia, which describes the complete absence of sperm in the ejaculate. In cases of OA, normal sperm production occurs in the testes, but the blockage in the epididymis, vas deferens or urethra prevents sperm from reaching the ejaculate [121–124]. Treatment options often include surgical correction of the blockage, which may allow sperm retrieval for ART such as in vitro fertilization (IVF). In NOA cases, the testes are unable to produce sperm, often due to underlying problems in spermatogenesis such as genetic abnormalities, hormonal imbalances, testicular infections or environmental factors. Treatment options are more challenging and may include hormone therapy, testicular sperm extraction (TESE) to retrieve sperm for ART or seeking alternative options such as sperm donation [125–127]. The distinction between OA and NOA is crucial for diagnosis and treatment planning. Diagnosis typically relies on a physical examination, semen analysis, hormone testing and possibly a testicular biopsy to differentiate between the two.

Liu et al. investigated the possible involvement of circ_0049356 in NOA using blood and semen samples. Using quantitative real-time PCR, they analyzed the expression levels of circ_0049356 and its host gene CARM1. Given the established role of circRNAs as miRNA sponges affecting gene expression, they predicted 5 miRNAs and 101 mRNAs as potential downstream targets of circ_0049356. Subsequent bioinformatics analysis of this predicted circRNA-miRNA-mRNA network revealed that the target mRNAs are associated with guanyl nucleotide exchange factor activity, GTP/GDP exchange, and regulation of the actin cytoskeleton, processes that are critical for cytoskeletal remodeling in germ cells during sperm production [128].

Another study using a high-throughput circRNA microarray provided interesting results, showing 368 circRNAs with decreased expression and 526 with increased expression in NOA patients. Among them, hsa_circRNA_0023313 showed dramatically increased expression in NOA, suggesting that it may play an important role in the control of spermatogenesis and serve as a potential biomarker for the detection and treatment of NOA [129].

In another study, a total of 37 881 circRNAs were found to be differentially expressed in the testes of NOA patients compared with healthy controls. hsa_circ_0137890, hsa_circ_0136298, and hsa_circ_0007273 were among the DEcircRNAs confirmed by Sanger sequencing. Notably, these circRNAs were mainly localized in the cytoplasm. Bioinformatics analyses showed that several miRNAs that could be paired with circRNAs have important roles in the spermatogenesis regulation [130].

### CircRNAs and sperm retrieval rate

In men with NOA, micro-TESE, a refined technique for sperm retrieval, has shown promise but still has a variable success rate. This variability highlights the importance of accurate markers to identify patients who have the best chance of benefiting from this procedure. For decades, researchers have analyzed various clinical and laboratory indices, from testicular volume and hormone levels to pathological data and surgical methods, in the hope of finding reliable predictors. However, the existing variables are not sufficient to accurately predict the outcome of micro-TESE in NOA patients. Studies are now investigating whether specific circRNAs could be the key to predicting the success of micro-TESE in NOA patients. In the meantime, studies have shown the difference in the expression level of hsa_circ_0000277, hsa_circ_0060394, hsa_circ_0007773 and circular RNA monoglyceride lipase (circ_MGLL) in positive/negative sperm retrieval outcomes [131, 132].

### CircRNAs and sertoli cell (SC)-only syndrome

SCs are specialized cells in the testes that play a crucial role in the spermatogenesis. They act as supporting and regulating elements and influence this process through various functions. They mediate the effect of hormones such as testosterone and FSH on the developing germ cells, secrete inhibin B, which regulates the release of FSH by the pituitary gland, and ingest and remove cell debris produced during sperm development (phagocytosis). SCs release the sperm cells into the lumen of the seminiferous tubules (spermiation) and control the movement of ions and proteins within the testis. SCs also form the blood-testis barrier, which separates the developing sperm from the bloodstream and other body fluids. This barrier also forms various compartments in the seminiferous tubules that supports the special environment required for sperm production. The proliferation of SCs takes place before puberty and is influenced by hormones such as FSH, activin and thyroxine. A disruption of one of these functions can significantly impair spermatogenesis and male fertility [133–135].

Sertoli cell-only syndrome (SCOS), characterized by the absence of germ cells in tubules is the most severe histological form of germ cell aplasia. While previous genetic explanations have focused on karyotype abnormalities and microdeletions of the Y chromosome, many cases of SCOS remain unexplained [89, 135–138]. Thanks to advances in the sequencing and microarray technology, the search for new genetic causes has gained momentum. Studies are using targeted gene sequencing for individual cases and whole-exome sequencing for familial cases, which has led to the identification of several SCOS-related genes. In addition, closer examination of the testicular transcriptome, proteome and epigenetics of SCOS patients is shedding new light on the molecular mechanisms underlying this disease.

A study by Zhu et al. investigated expression of circRNAs in SCOS. Using high-throughput analysis, they identified a striking pattern: 1594 circRNAs were expressed differently in SCOS testes compared with OA cases. Most importantly, most of these circRNAs were downregulated, suggesting that they normally play a crucial role in healthy sperm development but are disrupted in SCOS. Interestingly, these DEcircRNAs were primarily derived from protein-coding genes and spanned across all chromosomes, suggesting their possible involvement in regulating gene expression during sperm maturation. hsa_circRNA_101222, hsa_circRNA_001387, hsa_circRNA_001153, hsa_circRNA_101373 and hsa_circRNA_103864 were involved in biological processes such as regulation of the cell cycle and communication between cells, as well as sperm development. Overall, the research findings suggest that abnormally expressed circRNAs may contribute to the development of SCOS by impairing the function of SCs and the spermatogenic microenvironment [139].

In another study, the expression of hsa_circ_0000116 was found to be significantly higher in testicular tissue samples from NOA patients than in OA patients. In addition, hsa_circ_0000116 is abnormally expressed in different subtypes of NOA (SCOS, MA, HS), with the highest levels in SCOS patients. hsa_circ_0000116 is negatively correlated with sperm quality and positively correlated with FSH levels. High expression of hsa_circ_0000116 is associated with a lower testicular sperm retrieval success rate. Bioinformatics analysis suggests that hsa_circ_0000116 functions as a ceRNA for hsa-miR-449a and may influence fertility [140].

### CircRNAs and blood-testicular barrier

This barrier is produced by tight connections between the SCs and has crucial roles in the spermatogenesis. Zhang and colleagues found that DEcircRNAs are strongly associated with the tight junction signaling pathway. Two specific circRNAs, circRNA 1774 (from the *CDC42* locus) and circRNA 18,184 (from the *PTEN* locus), were less abundant in sexually mature testes. As the blood-testis barrier forms and matures, the proportion of SCs in the testes decreases. With fewer SCs, lower levels of tight junction-related proteins (and their associated circRNAs) are expected. The study also found that DE-circRNAs are enriched in the mTOR complex signaling pathway. For instance, circRNA 40,370 (from the *RICTOR* locus) was significantly more abundant in sexually mature testes. This may underscore the significance of the RICTOR/mTOR complex 2 signaling pathway for sperm development and SCs function and may influence the integrity of the blood-testis barrier and sperm production overall [95]. These research findings suggest that circRNAs may play a regulatory role in the development and maintenance of the blood-testicular barrier, a critical component for healthy sperm production. Understanding how these circRNAs function could provide insights into male fertility and potentially lead to new strategies for the diagnosis or treatment of male infertility.

### CircRNAs and asthenozoospermia

Asthenozoospermia, characterized by sluggish or immobile sperm, is a major obstacle for many couples trying to conceive. This condition is defined by low overall and progressive sperm motility (below 40% and 32% respectively) and impedes the ability of the sperm to reach and fertilize the oocytes. Sperms rely heavily on mitochondria, organelles concentrated in the center of their tail, to convert energy into movement. Further research suggests that other metabolic pathways may also play a role in this condition [141–143]. Despite advances, the exact molecular mechanisms underlying asthenozoospermia are still largely unclear. The complex interplay of various factors, likely involving both mitochondrial and alternative metabolic pathways, contributes to this puzzling condition.

Studies have identified several genes that may be associated with asthenozoospermia, including CRISP2, CATSPER1 and PATE1. Recent research has focused on the role of circRNAs in the regulation of these genes and their potential effects on sperm motility [144–148]. Francesco et al. identified three circRNAs (circTRIM2, circEPS15, circRERE) associated with the expression of CRISP2, CATSPER1 and PATE1, respectively [149]. These circRNAs were downregulated in low-motile spermatozoa (B spermatozoa) from patients with asthenozoospermia. In addition, oral amino acid supplementation, which is known to improve sperm motility, increased the expression of these circRNAs and their respective target genes in B spermatozoa. Their study also identified another circRNA (circSEPT10) involved in the regulation of CRISP2 expression [149]. These research results suggest that modulation of circRNA expression could be a potential approach to treat asthenozoospermia and improve male fertility.

In the field of animal studies, researchers investigated the possible role of circRNAs in influencing sperm motility in Yili geese [150]. They compared testicular tissue from geese with high and low sperm motility. They identified a total of 26,311 circRNAs and found 173 that differed significantly between the two groups (82 upregulated and 91 downregulated). The results showed that certain circRNAs are associated with variations in sperm motility. The analysis revealed that these circRNAs may influence genes involved in different biological processes. A network of interacting molecules was constructed, comprising 20 circRNAs, 18 miRNAs and 177 mRNAs. Nine important circRNAs were identified that may regulate sperm motility through interaction with miRNAs and subsequently influence mRNA expression. These research results suggest that circRNAs may play a crucial role in regulating sperm motility in Yili geese [150]. The identification of specific circRNAs and their interactions with other molecules could provide valuable insights into the mechanisms that control sperm motility at the molecular level. This information could be used to develop strategies to improve sperm quality and fertility in these geese.

The potential role of circRNAs in the reproductive system of yaks and cattleyaks was also investigated. Li et al. found 1,298 circRNAs in the epididymis of both species. A total of 137 of these circRNAs showed differences in expression between yaks and cattleyaks. Analysis revealed these circRNAs were mainly related to metabolic processes, DNA replication, recombination, repair, cell adhesion, growth and reproductive processes. This study highlights the potential involvement of circRNAs in yak and cattleyak reproductive function [151]. Understanding how these molecules differ between species and their specific functions could provide insights into male fertility and potential strategies for improving reproductive outcomes in these animals.

Table 1 shows cirCRNAs that are involved in male infertility.

**Table 1:** Some of circRNAs involved in male infertility

CircRNAs	Targeted miRNA	Gene	Host gene	Function	Refs
hsa_circ_0136298	miR-221	KIT	-	Mammalian male germ cells integrity	[130]
hsa_circ_0000116	miR-449a	-	MAN1A2	Autophagy and spermatogenesis	[140]
ssc_circ_1370	miR-28		FAM92A	promote ciliogenesis	[152]
hsa_circ_0136298 and hsa_circ_0007273	miR-762	-	-	Replication or apoptosis of Sertoli cells	[153]
Circ-MGLL	miR-1228, miR-1233, miR-149, and miR-924	-	-	Replication, apoptosis and differentiation of SC	[132, 154]
ssc_circ_0002			WDR7	semen quality	[155]
circ_0049356		CARM1	-	Dynamically restructuring the cytoskeleton of germ cells throughout spermatogenesis	[128]
ssc_circ_0954	miR-361-3p		DCDC2C	regulate the structure and movement of the sperm’s tail	[156–158]
hsa_circ_0023313	miR-372–3p	RAB-24 and USP-24	-	Autophagy, deubiquitination process and spermatogenesis	[159]
ssc_circ_0361	miR-26a	-	ACTL6A	Sperm motility	[160, 161]
hsa_circRNA_402130	let-7 miRNA family	LIN28A	-	Monitoring stem cell	[162]
ssc_circ_1219	miR-101	-	OSBPL9	Sperm motility	[162]
hsa_circRNA_101373	-	BCL6, FOXO1, and HMGA2	-	Cell differentiation and stem cell integrity in SC	[164]
ssc_circ_1352	miR-26a	PTEN and PMAIP1	CAGE1	Acrosomal characteristics	[165]
hsa_circRNA_0023313	hsa-miR-520d-3p, hsa-miR-373-3p, hsa-miR-372-3p, has-miR- 302c-3p and hsa-miR-130b-5p	-	-	Control of spermatogenesis via FoxO and AMPK signaling pathway	[129]
ssc_circ_1454	miR-16 and miR-423-5p	-	MTHFD2L	Sperm quality and male fertility	[166]
ssc_circ_0345	miR-423-5p	-	SLC5A10	Spermatid differentiation	[167]
ssc_circ_0780	-	-	LRGUK	Sperm motility	[156]
ssc_circ_1532	miR-99b	-	SPATA19	Sperm motility	[168, 169]
ssc_circ_1458	-	-	LRBA	Sperm motility	[156]
ssc_circ_0839	-	-	PAIP2	Spermatogenesis	[170]
ssc_circ_1321	-	-	PAPOLA	Sperm motility	[156]
ssc_circ_0823	-	-	CAMK4	Sperm motility	[171]
circTRIM2	hsa-miR6721–5p	CATSPER1	-	Asthenozoospermia	[149]
circEPS15	hsa-miR-138–5p	PATE1	-	Asthenozoospermia	169
circRERE	hsa-miR-27b	CRISP2	-	Asthenozoospermia	[149]
circCIT, circUSP54, circTRMT2B and circTADA2A	-	-	-	Weak spermatozoa	[93, 172]
circNAPEPLD	-	-	-	Sperm quality	[173]
circRNA_06790		CTNNB1		Spermatogonia differentiation	[174]
circ_0002265	miR-122-5p	PAFAH1B2	-	Gonadal development	[175]
CircADAM10	mmu-miR-670-3p, mmu-miR-509-5p, mmu-miR-1903, mmu-miR-5106, and mmu-miR-7220-3p	-	-	Oxidative stress and oligoasthenozoospermia	[117, 176, 177]
circPCSK6	mmu-miR-7033-5p, mmu-miR-667-5p, mmu-miR-191-3p, mmu-miR-3073a-3p, and mmu-miR-6989-3p	-	-	ROS production and spermatogenesis failure	[117, 178, 179]
hsa-circRNA-101,373	-	BCL6, FOXO1 and HMGA2	-	Apoptosis and spermatogenesis	[139]
circRNA9244 and circRNA 10,187	-	SMARCA5	-	Testicular development	[180]
circRNAs-6682	-	NSD1	-	Androgen receptor binding	[180]
circRNAs-10,979	-	POC1A	-	Function of spermatogonial stem cell and Sertoli cells	[180]
circRNAs-18,456		TDRD1	-	Germ cell development	[180]

### Exosomal circRNAs and male infertility

Exosomes, tiny extracellular vesicles ~ 30–150 nanometers, represent a diverse population of messengers released by different cells throughout our body fluids and tissue environments [181–184]. They function as crucial information carriers, shuttling signaling molecules like ncRNA (miRNA, circRNA, lncRNA), lipids, proteins, and biomolecules between cells. Exosomes as miniature envelopes, meticulously assembled within cells by the inward budding of late endosomes, compartments responsible for sorting and recycling cellular material. Once packaged, they are released into the extracellular space, ready to deliver their cargo to recipient cells. Importantly, the specific molecules carried by each exosome vary depending on its originating cell, creating a unique communication system tailored to specific functions. The unique composition, quality, and quantity of each exosome reflect its cellular origin [185–189]. This makes them valuable biomarkers for diagnosing and predicting the course of various diseases, ranging from cancer [190] to infertility [191].

Exosomes offer a promising non-invasive approach to diagnosing male infertility, particularly in azoospermia cases. Analyzing the specific types and levels of these ncRNAs within seminal fluid can potentially distinguish between two main types of azoospermia (OA and NOA) [192–195]. This crucial information helps physicians understand the underlying cause of sperm absence, be it a physical blockage or a production issue within the testes. Ultimately, such insights pave the way for determining the most effective treatment, including identifying hidden sperm in the testes for retrieval in select cases.

### Promising advances in genetics and male infertility research

Despite the significant impact of male infertility (it accounts for 50% of infertility cases and is the sole cause in 20% of cases), treatment options for men lag far behind those for women (≥ 15 years). Although there are proven strategies to improve male reproductive potential, 27% of male partners of couples facing infertility receive no treatment at all [196, 197].

Several exciting areas of research have the potential to revolutionize the way we diagnose and treat male infertility. For instance, artificial intelligence and machine learning can enhance diagnostic processes through automated analyses of medical images and data, increase accuracy, detect subtle patterns often overlooked by human experts, and provide tailored treatment recommendations based on individual patient data [198, 199]. These methods can also facilitate identification of potential circRNAs contributing to the pathogenesis of male infertility.

Next-generation sequencing and other “omics” techniques are pinpointing the underlying genetic causes of male infertility. These techniques have various implications in understanding developmental defects, identifying mutations and copy number variations that disrupt reproductive system development, expanding the catalog of genes linked to male infertility, improving diagnosis and potential targets for future therapies, and mapping gene expression patterns, providing unprecedented insights into sperm development and function [135, 200, 201].

## Conclusion

This research highlights the exciting potential of circRNAs as key players in male fertility. They appear to influence how germ cells, the precursors to sperm, grow and divide during the sperm production process (spermatogenesis). This suggests that malfunctions in circRNA activity could contribute to male infertility issues stemming from abnormalities in spermatogenesis.

Identification of the role of circRNAs in male infertility would pave the way for design of novel therapies, particularly methods to achieve in vitro spermatogenesis (creating sperm in the lab) and regenerate sperm production in vivo (e.g., after cancer treatment). The rapid pace of research is driving a deeper understanding of the causes of male infertility. This will lead to improved diagnostics, more precise identification of infertility causes and associated health risks, personalized medicine, and tailored treatments based on individual genetic profiles.

## Data Availability

No datasets were generated or analysed during the current study.
